# Changes in soil carbon mineralization related to earthworm activity depend on the time since inoculation and their density in soil

**DOI:** 10.1038/s41598-022-17855-z

**Published:** 2022-08-10

**Authors:** Patricia Garnier, David Makowski, Mickael Hedde, Michel Bertrand

**Affiliations:** 1 Université Paris-Saclay, INRAE, AgroParisTech, UMR Ecosys, 91120 Palaiseau, France; 2 Université Paris-Saclay, INRAE, AgroParisTech, UMR MIA 518, 91120 Palaiseau, France; 3 INRAE, UMR Eco & Sols, 2 Place Viala, 34070 Montpellier, France; 4 Université Paris-Saclay, INRAE, AgroParisTech, UMR AGRONOMIE, 91120 Palaiseau, France

**Keywords:** Ecology, Biogeochemistry, Ecology, Environmental sciences

## Abstract

Earthworms play a key role in soil carbon mineralization, but their effect is highly uncertain and suspected to vary as a function of several factors, particularly the earthworm density and time from earthworm inoculation. We conducted a meta-analysis considering these factors based on 42 experiments comparing carbon mineralization in the absence and presence of earthworms at different times. The results reveal an average carbon mineralization increase of 24% (sd 41%) in the presence of earthworms with an initial median earthworm density of 1.95 mg/g soil DM (Dry Mass) (sd 48%). We show that carbon mineralization due to earthworms was related to their density and time from inoculation. From a simple regression model using these two variables, the estimated impact of earthworms on carbon mineralization was 20% increase from 0 to 60 days and 14% decrease at day 350 for a density of worms commonly found in soils (0.5 mg/g soil DM). Finally, we proposed a simple equation that could be used in organic matter decomposition models that do not take macrofauna into account.

## Introduction

Containing a total of 1580 Gt of carbon globally, soils are crucial carbon pools for mitigating climate change^1^. Changes in the carbon stock depend on the mineralization/sequestration balance, which is often calculated with models. However, current mineralization models are built without taking macrofauna into account^2^. To simulate and assess the impacts of climate and/or land use changes on biogeochemical fluxes, it is imperative to better integrate macrofauna into models since earthworms play key roles in carbon mineralization^3^. While global earthworm distribution maps are now available^4^, incorporating earthworms into C mineralization models is still challenging.

In the literature, the effects of earthworms on the organic carbon mineralization are contradictory because of the multiple physical and chemical interactions between earthworms and soil^5,6^. On the one hand, some studies have shown that earthworms enhance carbon mineralization because they create close contact between organic matter and microorganisms in their casts or burrows, thus facilitating microbial activities^7^. On the other hand, some authors have shown that the activity of earthworms promotes the formation of aggregates that physically protect organic matter and allow its stabilisation^8^. In published experiments, the great variety of materials (earthworm types, soil types and added organic matter types), and experimental conditions (experimental durations, climate conditions, microcosm sizes, etc.) preclude the emergence of shared patterns. Thus, a thorough and accurate analysis of the literature is required to relate the explanatory variables of earthworm-induced CO_2_ emissions. Based on a meta-analysis, the study^9^ found that earthworms significantly increase CO_2_ emissions up to 200 days after their introduction in soil. They^9^ recommended considering longer experimental duration when assessing the impacts of earthworms on CO_2_ emissions to analyse their impact after 200 days. The earthworm-induced CO_2_ emissions is also to be strongly dependent on several other factors than time. A previous study^10^ also found that the worm density affected earthworm-induced CO_2_ emissions. Mathematical models considering earthworm carbon mineralization should be proposed based on meta-analyse in order to improve models of organic matter decomposition in soils.

Herein, we focused on the impact of earthworms (EWs) on CO_2_ emissions by considering the effects of experiment durations and worm densities. The novelty of our paper is to propose an equation to take organic carbon mineralization from earthworms into account. We developed a meta-regression model based on 42 experiments including at least three pair of mineralization observations with and without earthworms and then use this model to provide new estimates of the effects of earthworms on CO_2_ emissions. We considered experiments duration from 16 to 378 days. Unlike other meta-analysis of literature, we studied here the statistical variability between treatments. We compared the effect of time, either on all data considered as independent, or on the data of each treatment considered as dependant.

## Results

### Effects of time since earthworms inoculation on CO_2_ emissions

**Figure 1:**
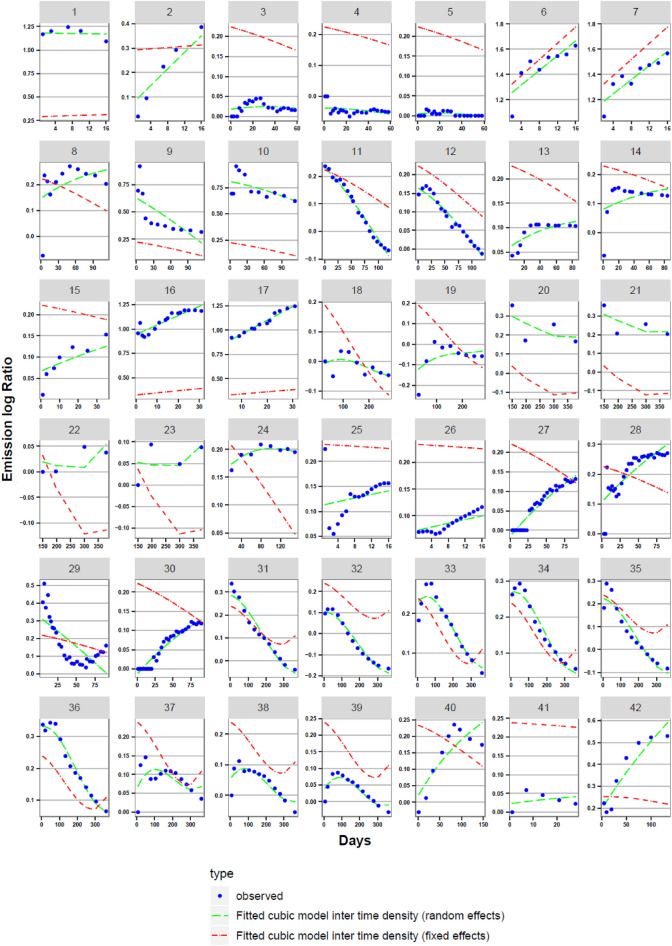
Evolution of the log ratio of CO_2_ emissions with and without EW with time in the 42 experimental situations; comparison between experimental data (blue curve) and both mathematical models: one model with fixed temporal effects (red curve) and one model with the random temporal effects (green curve). The statistical parameters of both models are given in Table 2. The corresponding publication for each figure is: Figs. 1, 2^18^, Figs. 3–5^31^, Figs. 6, 7^32^, Figs. 8–10^7^, Figs. 11, 12^19^, Figs. 13, 14^33^, Fig. 15^30^, Figs. 16, 17^34^, Figs. 18, 19^27^, Figs. 20–23^22^, Fig. 24^35^, Figs. 25, 26^17^, Figs. 27–30^28^, Figs. 31–39^36^, Fig. 40^16^, Fig. 41^29^, Fig. 42^37^.

The effects of earthworms on CO_2_ emissions strongly depend on the amount time that has passed after their inoculation. We used 546 pairs of cumulative CO_2_ emission obtained with and without EWs from 42 treatments and extracted from 17 articles (the residual frequency histogram is presented in Fig. SI1, the Skewness normality index of 0.325 is close to 0 and indicates that normality is satisfied). We found that EW presence increases soil CO_2_ emissions by 24% (sd = 41%) on average compared to controls without EW, but the effect of earthworm presence is highly dependent on the timing of measurement after earthworm introduction. The experimental curves of the log ratio of CO_2_ emissions with and without EW exhibit three types of aspects (blue circle curves in Fig. 1):

(i) Dynamics showing a fast increase in emissions followed by a slow decrease (21 cases out of 42; see, e.g., #33). This reveals a more rapid increase in mineralization followed by a slower decrease in the presence of earthworms. This type of curve is generally obtained for experiments lasting more than 100 days. These curves can reach negative values at the end of the experiments, indicating that at that time, the presence of earthworms led to carbon sequestration (this was observed in one-third of studies, e.g., #12 and #35).
(ii) Dynamics showing a continuous positive trend or a positive trend followed by a plateau (15 cases out of 42; see, e.g., #13). This means that the presence of earthworms led to greater mineralization, which increased over time with the stabilisation of this mineralization rate. This type of curve was obtained mainly in short-duration experiments (with maximum durations < 40–80 days).
(iii) Dynamics characterised by a decreasing trend (6 cases out of 42; see, e.g., #11). The presence of earthworms initially led to additional mineralization that decreased over time. This type of curve was recorded in experiments generally lasting more than 90 days (e.g., #31).

### Effects of earthworm density and experiment duration time on CO_2_ emissions

**Figure 2:**
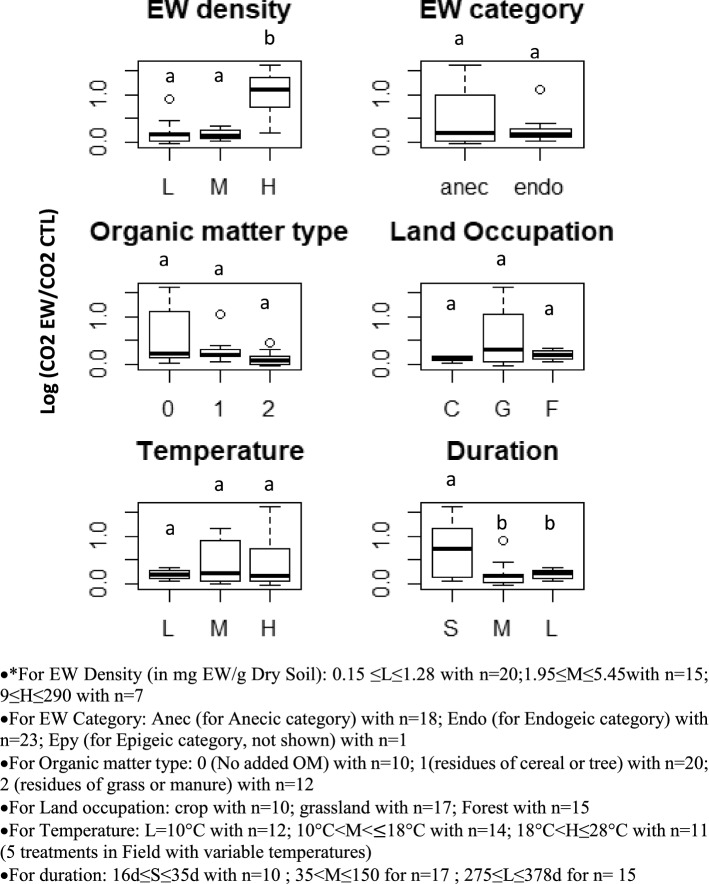
Impact of the factors on the logarithm ratio of CO_2_ emissions with and without EW. To build the box plot, each of the factors was split into 2 or 3 intervals*. All the experiments in the database measured carbon mineralization during at least the 15–21 day interval. We therefore calculated the log ratio emission between Days 15 and 21 according to the treatments.

After finding that the “time” variable had a great influence on carbon mineralization by earthworms, we investigated whether other factors could also have an influence. We selected factors for which we had information for all experiments. These factors are the density and category of earthworms, type of organic matter present in the soil, land use, temperature, and final duration of the experiment from inoculation of the earthworms. To study the influence of these factors, we selected a time period for which we had a maximum of CO_2_ measurements for each of the 42 treatments (between 15 and 21 days). Indeed, we wanted both to free ourselves from the effect of the “time” variable and for each treatment to be represented only once. We then created box plots of the log ratio values of CO_2_ emissions with and without EW for each of the factors, as shown in Fig. 2.

We observe that almost all the log ratios of CO_2_ emissions are positive, indicating again that treatments with EW emitted more CO_2_ than treatments without EW. At 15–21 days, the mean value of the log ratio of CO_2_ emissions was 0.334, indicating that treatments with EW emitted 39.7% more CO_2_ than treatments without EW. This value, calculated at only one date close after inoculation, is higher than the values found previously for all dates because the earthworm-induced CO_2_ emissions generally decrease over time (21 + 6 curves out of 42 in Fig. 1).

Figure 2 shows that CO_2_ emissions are mostly affected by the earthworm density and experiment duration. Indeed, the highest mean log ratio of CO_2_ emissions was obtained for the highest density of earthworms and the lowest experimental duration. Indeed, we observe significant differences in the log ratio emission between the groups, with p values of 3.21.10^–7^ for the EW density and 0.00139 for the experiment duration. The treatments with high EW densities (> 9 mg EW/g DM soil) show much higher mineralization than the treatments with lower EW densities. The experiments with short durations (< 35 days) show both a much higher mean mineralization and high variability. For the other factors, we did not observe any significant differences in the log ratio emission between the groups but we observed that the variability in the log-ratio emissions can be very high. For the EW category factor, the Anecic category shows a much higher variability of the log ratio emission than the endogeic category, for example.

### Link between earthworm density and experimental duration

**Figure 3:**
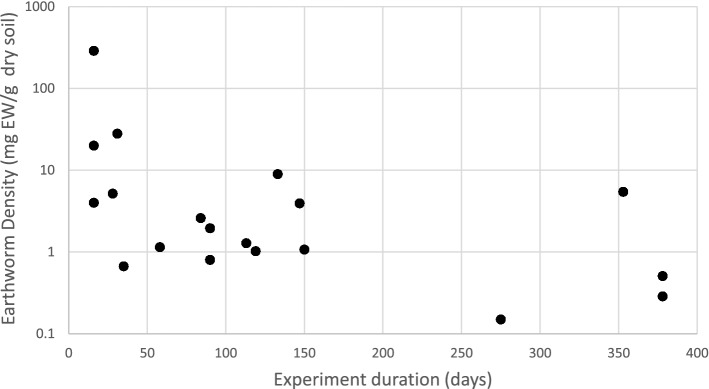
Earthworm density according to the duration of the experiments. The EW density distribution is as follows: Min: 0.15 mg EW/g dry, 1st quartile: EW density = 1.028 mg EW/g dry soil, 2nd quartile: EW density = 1.95 mg EW/g dry soil, 3rd quartile: EW density = 5.45 mg EW/g dry soil, max = 290 mg EW/g dry.

Researchers adjusted the earthworm density to the experimental duration. From our database, we found that earthworm densities (with a mean of 12.6 mg EW/g DM soil and a median of 1.95 mg EW g/g DM soil, sd of 48 g/g DM) were not distributed randomly with the experiment duration (Fig. 3). The EW densities are higher for short-duration experiments (< 50 days) with a mean of 57.97 mg EW/g DM soil than for experiments with longer durations (> 50 days) with a mean of 2.14 mg EW/g DM soil.

### Selection of models to simulate carbon mineralization from earthworms

We tested different models to better understand the influence of factors explaining the carbon mineralisation. The best model considers the “time (after EW inoculation in soil)” and “earthworm density” variables. To determine which variables best explain the C mineralization from EW, we tested different statistical models on our dataset of log-ratio CO_2_ emissions. The data were fitted to different model functions of time, assuming that the time variable can be either fixed or random. In the temporal-fixed-effect models, all observations are considered independent and time-dependent. In the temporal-random-effects models, each treatment is considered independent, but the observations of the same treatment are time-dependent. Different time-dependent models, with either fixed or random effects, were compared, ranging from linear to quadratic and cubic models. For the variables other than time, we considered a fixed-effect model. The statistical parameters AIC and BIC were used to evaluate whether the model fit the dataset well.

Including time as a factor significantly improves the models describing the effect of earthworm presence on carbon mineralization (with AIC decreasing from − 897 without time to approximately − 1200 with time for random-effect models, see Table 1). The random-temporal-effect models allow a much better simulation of the log ratio emission CO_2_ curves than the fixed-temporal-effect models (− 1200 vs. 300 of AIC and BIC). The cubic model (AIC of − 1263 for random-effect models) appears slightly better than the linear and quadratic models (AIC of − 1255, − 1256 for random-effect models). Additionally, the density of the earthworms is the only explanatory variable that makes a noticeable improvement of the simulation, with fixed-effects for this variable (with AIC of − 1285 versus AICs between − 1105 and − 1268 for the time random-effect model). Finally, the best model includes the interaction between the earthworm density and time (AIC of − 1360 for the random-temporal-effect model).

**Table 1:** Comparison between different mathematical models of the logarithm of the ratio between CO_2_ and EW and CO_2_ in CTL using different explicative variables. The comparison is based on the statistical criteria AIC (Akaike Information Criterion) and BIC (Bayesian Information Criterion). Lower AIC and BIC values indicate a better fit with the experimental data. The models with random temporal effects consider that data belonging to the same treatments are not independent, and the models with fixed temporal effects consider each datapoint to be independent from the others.

	Time fixed-effect		Time random-effect	
	AIC	BIC	AIC	BIC
**Time model**				
Without time effect	404	412	− 897	− 884
Linear model with time	366	379	− 1255	− 1234
Quadratic model with time	358	375	− 1256	− 1226
Cubic model with time	357	379	− 1263	− 1225
**+ Co-variable (fixed-effect)**				
+ EW density	47	73	− 1285	− 1242
+ EW category	345	371	− 1264	− 1216
+ OM type	281	311	− 1261	− 1205
+ Temperature	365	390	− 1105	− 1059
+ Land occupation	331	357	− 1268	− 1225
**Interaction with density**				
Time density interaction	36	66	− 1360	− 1304

Therefore, the cubic model including ‘time’ with random effects, ‘earthworm density’ with fixed effects, and their interaction (Table 2) better fit the observed data than the model including ‘time’ with fixed effects model (see Fig. 1). It can adapt the log ratio of the CO_2_ emission curve shape to each experiment, while the fixed-effect model leads to a decreasing line that depends on the earthworm density. Additionally, this temporally random model has p values lower than 0.05, while the fixed-effect model has p values sometimes greater than 0.05 (Table 2).

**Table 2:** The mean value standard error and its statistical significance for the cubic mixed-temporal-effect model + EW density (random temporal effects and fixed EW density effects) and for the cubic fixed-temporal-effect model + EW density. The equation of the model is as follows: Ln (CO_2_^EW^/CO_2_^CTL^) = a** + **bxTime + cxEW_Density + dxTime^2^ + exTime^3^ + fx(Time/EW_Density).

	Value	Std error	DF	t-value	p-value
**Cubic time random-effect + EW density fixed-effect model**					
a: Intercept	0.1828	0.0458	500	3.99	0.0001
b: Time	0.00017	0.00044	500	2.09	0.0371
c: density	0.003649	0.00071	40	5.09	0.00001
d: Time2	− 6.81E−6	1.90E−6	500	− 3.91	0.0001
e: Time3	1.17E−08	3.41E−09	500	3.64	0.0003
f: Time/density	0.00022	0.00001	500	9.62	2.37E−6
**Cubic time fixed-effect + EW density fixed-effect model**					
a: Intercept	0.22	0.0265	500	9.69	< 2E−16
b: Time	− 0.000919	0.000794	500	− 1.101	0.242
c: Density	0.00359	3.64E−4	40	9.839	< 2E−16
d: Time2	− 4.13E−6	− 6.06E−6	500	− 0.131	0.496
e: Time3	1.09E−08	1.202E−08	500	0.377	0.363
f: Time/density	0.0046	0.000227	500	20.408	0.000274

**Figure 4:**
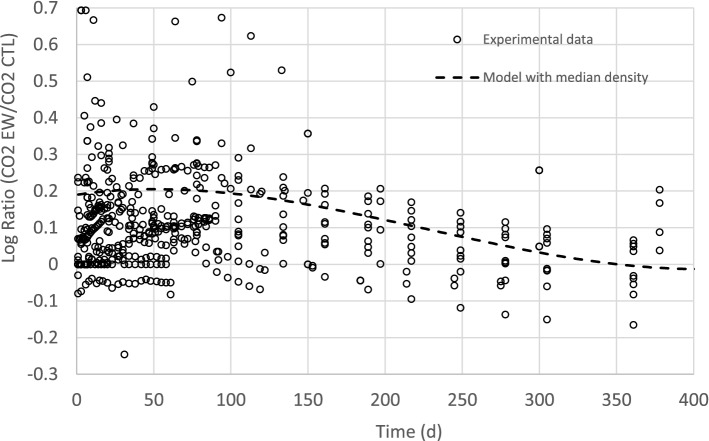
Comparison between all the experimental data and the simulated results of the logarithm ratio of CO_2_ emissions with EW and without EW as a function of time and earthworm density for the 42 experiments. Simulations were carried out with the best model based on random temporal effects and fixed EW density effects (see the equation, parameters and statistical parameters given in Table 2). The median EW density of our experimental set (median Exp. = 1.95 mg EW/g dry soil) was used.

We plotted the full dataset (Fig. 4) and the curve calculated with our model (equation given in Table 2) by taking the median EW density values (1.95 mg EW/g soil DM, etype = 48). The modelling curve passes through the experimental data. The curve first increases until day 60 with a maximum increase in carbon mineralization from the EW treatment of 22.8% compared to the control treatment; the curve then decreases until it becomes negative at day 350. The estimated impact of earthworms on mineralization ranges from 20% (at day 60) and − 11% (at day 350) if the model uses the worm density of 0.5 mg/g soil DM commonly found in real conditions in the literature^11,12^.

## Discussion

We found that EWs has a positive effect on carbon mineralization. We know that EWs actively participate in the incorporation of organic matter into the soil^5^. EWs also put the organic matter in close contact with the soil and its microorganisms in their casts. These situations are favourable under carbon mineralization because organic matter incorporated into the soil mineralizes faster than if it is left at the soil surface^13^ and the fragmented organic matter that is in closer contact with soil also mineralizes faster^14,15^. Microbial activity increases in the presence of EWs^16^. C^13^ labelling technology applied to fresh organic materials has shown that EWs increased the mineralization of the carbon in straw compared to the treatment without EWs^17^. EWs also generate a priming effect by stimulating the mineralization of soil organic matter^18^.

In contrast to the meta-analysis conducted in a previous study^9^ in which each observation was considered to be independent, we tested a statistical model that took into account the time effect on intertreatment variability. We show with our dataset that the random-effect models allow much better representations of the data than the models with fixed temporal effects, as it was used in^9^.

In Fig. 1, we found that the majority of the log-ratio emission curves decrease with time (21 + 6 on 42). Rising curves (15/42) are obtained mainly for short-duration experiments. For example^19^, as found in curves #11 and #12, during the first 30 days, earthworms significantly increased CO_2_ emissions by 16% to 28%. In contrast, significantly lower CO_2_ emission rates were measured after 80 days. The best model that adjusts the log ratio of CO_2_ emissions (a mixed-effect model) decreases over time if realistic EW density values are considered. In their review, Lubbers et al.^9^ confirmed that earthworm-induced CO_2_ emissions decrease over time for experiments with durations longer than 200 days. EWs ingest OM and mix it with the soil and binding agents in their gut. This mixture is excreted as casts. First, there is a mineralisation because the organic matter is in close contact with the microorganisms. Then, water-stable aggregates in which SOM is physically protected^20^ may be built over time in the casts. EWs form a significant pool of protected C (22% of the fresh residue carbon) in microaggregates after a few days of incubation^7^ for optimal conditions. We also found that 7 curves have a log-ratio CO_2_ emissions lower than 0 at the end of the experiments, indicating less CO_2_ emissions with EWs. In these cases, EWs redistributed organic carbon from less stable pools to more stable aggregates where organic matter is associated with minerals by the microbial cell wall and extracellular polymeric substances^6^.

In most of the experiments included in our dataset, earthworms were inoculated at the beginning of the experiment, which is different from reality where earthworms are already present and have already built their galleries. However, these laboratory experiments in which earthworms are initially artificially added to soils, can represent the real field conditions. Indeed, there can be significant fluctuations over time in the presence and activity of earthworms in the field. Earthworms are not very active during winter when temperatures are low or during summer when soils are too dry. In contrast, they can become active again at the beginning of spring and when organic matter is added to the soil^21^. To study the effect of earthworms over time, it is important to have enough experiments with long duration (longer than the plant-growing season). Most experiments are short enough to avoid dealing with mortality (< 100–200 days). Longer-term experiments (longer than 200 days) are more realistic. However, for these long durations, problems associated with earthworm mortality can arise. Thus, earthworms must be progressively re-inoculated as in^22^. If they are not, the density can decrease during the experiments and may distort the results. In addition, the container must be large enough for them to reproduce.

We showed that among the explanatory variables, the EW density was an important factor explaining the high CO_2_ emissions by earthworms (Fig. 1 and Table S2). However, the high-density group shown in Fig. 1 (> 5 mg EW/g dry soil) is not realistic compared to the measured field data. A mean earthworm density value of approximately 0.5 mg EW/g was registered in the field^11,12^ while we obtained a mean value of 12.6 mg EW/G and a median of 1.95 mg/g for all 42 treatments. Most of the experiments mentioned in our article refer to experiments in laboratory columns where earthworms were inoculated with occasionally very high densities to test their effect. We observed that higher densities were used in shorter experiments. There are two main reasons for this. First, maintaining high earthworm densities over long periods in micro- or mesocosms may be difficult because of their mortality. Second, it is likely that the researchers designed more realistic experiments over long time frames while amplifying processes in short-term experiments. The maximal earthworm density registered in the field can reach 5 mg EW/g^23^. Earthworm densities greater than this value may not be realistic. A few short-term experiments overpassed that threshold and mainly led to a the log-ratio CO_2_ emission curves rising without reaching plateaus (e.g., #1, #7 or #17 in Fig. 1). The differences in density measured in the field according to land occupation or soil tillage may be important but small when compared to the variations contained in our dataset. Grassland has an EW density 2 times higher than that of arable land^24^. In no-tillage fields, 2 to 9 times more EWs were registered than in fields with conventional tillage^22^.

In the last decade, attempts have been made to model the effects of EW on carbon dynamics^25^. In both studies, the authors considered a 2-phase model, an active mineralization phase that corresponds to mineralization in the gut or in fresh casts and a carbon-stabilisation phase in ageing casts that could lead to carbon sequestration^25^. We have proposed a model (with random temporal effects and fixed EW density effects in its interaction with time). When using the median EW density value calculated from our dataset, the earthworm-induced CO_2_ emissions follow this trend with an increase in carbon mineralization at short times following the introduction of EWs and decreasing carbon mineralization at longer times. In future studies, our earthworm-induced CO_2_ emission model could be tested under real field conditions starting at the beginning of spring and using the earthworm density evolution either measured in the field or simulated using population models (such as that proposed in^26^).

## Methods

### Literature review

Articles were selected from a literature review conducted with the ISI Web of Knowledge using the “All Databases” option to select studies in which SOM mineralization was measured under paired conditions with and without EWs. The literature search was carried out with the keywords included in the following search equation: cf SI1. The first terms were designed to select papers dealing with organic matter, and the second terms were designed to select papers dealing with earthworms. A total of 83 articles emerged from this first selection.

### Paper selection and data extraction

The references were selected according to the following criteria: (i) each paper had at least 3 mineralized carbon measurement points over time, (ii) provided information on explanatory variables such as the earthworm density, earthworm type, moisture content, temperature, soil type and organic matter type, (iii) did not process “*Eisenia fetida*”, a manure worm, to restrict the study to only worms that are present in the agricultural soils. An initial selection was made by analysing titles and abstracts. If they included relevant criteria, then the full text was examined. A total of 17 articles with 42 treatments emerged from this second screening. The carbon mineralization data were then extracted if they were in tables or using Datathief software if they were in graphs (https://datathief.org/) (see Table S1).

### Build the database

A table was then created with 18 columns. Table S1 summarises the database. It includes (i) generalities such as the paper number, names of the authors, treatment numbers, and type of experiment (laboratory of field), (ii) explanatory variables such as the category and species of earthworm, type of soil, land occupation, temperature, EW density, type of added organic matter and its C:N, type of treatment (with or without EW), duration, and number of replicates. Finally, the last column of the file contained the cumulative CO_2_ data from time 0.

The database presentation (Table S1) presents the database. The 17 publications correspond to 42 treatments in which the authors often studied the effect of adding organic matter or the effect of the organic matter quality. We obtained 542 pairs of CO2 emission (with EW and without EW for each treatment) at different times. Some publications examined the effect of earthworm categories/species^22,27,28^. Most of the experiments were carried out in the laboratory except in three articles that took place in the field^16,19,27^. We found only one treatment that studied the effect of epigeic worms^29^, while the other studies are shared between endogeic (the majority; 23/42) and Anecic worms. For laboratory experiments, the temperatures ranged between 10 and 28 °C. There were only 2 experiments with conditions beyond 20 °C^18,30^ because earthworms cannot withstand high temperatures. The duration of the experiments ranged between 16 and 378 days. The earthworm densities were in the range of 0.15–290 mg EW/g dry soil. The land cover for in situ experiments or at the time of soil sampling essentially corresponded to grasslands (17/42) versus 15/42 for forests and 10/42 for croplands. It was very difficult to use the variable “humidity” because the units were very variable from one study to another, and the data available in the articles did not allow us to unify the measurements. Therefore, this variable was not used in the analysis.

### Response variable

The response variable was calculated as the cumulative carbon mineralization ratio between experiments with earthworms and without earthworms. The log ratio of the CO_2_ emissions, F, was used as an effective size metric to normalise the data for the meta-analysis:

$$ {\text{F}} = {\text{Ln}}\,({\text{CO}}_2{\text{-EW/CO}}_{2}{\text{-CTL}}), $$

where CO_2_-EW is the cumulative CO_2_ with earthworms and CO_2_-CTL is the cumulative CO_2_ without earthworms. The F(t) curves are plotted in Fig. 1.

### Statistical analysis

The data were fitted to different statistical models of the response variable (log ratio of CO_2_ emissions) as a function of time, assuming that the temporal variable was either fixed or random. In the fixed-temporal-effect models, all observations from all treatments were polled and were considered to be independent. In the random-temporal-effects models, all observations from the same treatment were polled. In these models, treatments are considered to be independent, but successive observations of the same treatment are dependent. For the variables other than time, we considered fixed-effect models.

Different time-dependent models with either fixed or random effects were compared, ranging from linear to quadratic and cubic models. Models with fixed temporal effects were obtained by parameterizing the models on all the datasets without distinction among treatments, as was conducted in a previous meta-anaylsis^9^. The models with random temporal effects were obtained by parameterizing each treatment independently of one another while considering that the data of the same treatment were not independent. The mean values of the parameters were then calculated. Other explanatory variables were added to both models, such as the EW density, EW type, type of organic matter, land occupation and temperature. The model parameters were estimated by restricted maximum likelihood using the lme and glm functions from the nlme and stats packages (R v.3.1.2). The models were ranked according to two statistical criteria, the Akaike Information Criterion (AIC) and the Bayesian Information Criterion (BIC); a lower AIC/BIC value corresponds to a better model.

## Supplementary Information

Supplementary Information.

## Data Availability

https://www6.versailles-grignon.inrae.fr/ecosys/Personnes/Personnel-par-ordre-alphabetique/G/Garnier-P/Scientific-Reports-Soil-carbon-mineralization-related-to-earthworm-activity.
