# Supplementary material for: Changes in soil carbon mineralization related to earthworm activity depend on the time since inoculation and their density in soil
Source: Sci Rep. 2022 Aug 10;12:13616. doi: 10.1038/s41598-022-17855-z (PMC9365797; doi:10.1038/s41598-022-17855-z)

**Supplementary information**

SI 1

The equation used for searching the literature is shown here; the he first terms were designed to select papers dealing with organic matter, and the second terms were designed to select papers dealing with earthworms. A total of 83 articles emerged from this first selection.

(Carbon* OR minerali?ation* OR "organic matter"* OR "CO2" OR Respiration OR earthworm* ) AND ("earthworm*" OR "worm*" OR "lumbricus*" OR "Macrofauna" OR "Lumbricus terrestris" OR "Eisenia foetida" OR "Eisenia Andrei" OR "Lumbricus rubellus" OR "Eudrilus eugeniae" OR "Aporrectodea trapezoides" OR "Aporrectodea tuber-culate" OR "Aporrectodea turgida" OR "Eisenia hortensis" OR "Dendrobaena veneta" OR "Dendrobaena subrubicundus" OR "Eisenia eiseni" OR "Lumbricus rubellus" OR "Lumbricus castaneus" OR "Satchellius mammalis" OR "Dendrobaena octaedra" OR "Aporrectodea caliginosa" OR "Allolobophora rosea" OR "Allolobophora icterica" OR "Allolobophora chlorotica" OR "Octolasium cyaneum" OR "Aporrectodea giardi" OR "Aporrectodea longa" OR "Lumbricus rubellus" OR "Octolasium lacteum" OR "Pontoscolex corethrurus") AND (soil*)

Table S1: Short descriptions of the references and treatments included in the analysis (17 references and 42 treatments)

| References  and **Treatment number** | Laboratory/field | Category of EWs and Species (*) | Type of Soil | Land occupation | Temperature (°C) | Humidity | Density  (mg_EW/g_Dry soil) | Type of added OM | Number of treatments | Time (days) |
| --- | --- | --- | --- | --- | --- | --- | --- | --- | --- | --- |
| 1. Bernard et al. (2012) **1,2** | Laboratory | Endogeic  Sp1 | Ferralsol | Grassland | 28 | 100% of WHC | 20 | Wheat straw | 2 - with and without straw | 16 |
| 2. Bertora et al. (2007) **3,4,5** | Laboratory | Anecic  Sp2 | Typic fluvaquent | Grassland | 20 |  | 1.15 | Grass | 3 soil water contents | 58 |
| 3. Binet et al. (1998) **6,7** | Laboratory | Anecic  Sp3 | Silty Soil (Rennes) | Grassland | 20 | Θg=0.23 | 290 | 0 | 2 types of measurement | 16 |
| 4. Bohlen et al. (1995) **8,9,10** | Laboratory | Anecic  Sp3 + Sp4 | Mesic Fragiudalf | Grassland | 15 | 75% of water wight | 1.28 | Vetch residue  Cow manure  Granular N | 3organic matters | 112 |
| 5. Borken et al. (2000) **11,12** | Mesocosm in Field | Anecic  Sp3 | Acidic dystric Cambisol | Forest | [13-23] | [-15,-2] kPa | 1.028 | Beech litter | 2- with and without limed | 120 |
| 6. Butenschoen et al. (2007) **13,14** | Laboratory | Endogeic  Sp 5 | Mollisol, Haplic Chernozem Loam | Crops | 20 | θg=0.227 | 2.6 | Rye leaves | 2 - EW with and without contact to the leaves | 84 |
| 7. Chapuis Lardy et al. (2010) **15** | Laboratory | Endogeic  Sp1 | Clayey Andic Dystrustept or Ferralsol | Grassland | 25 | 40% of water weight | 0.66 | 0 | 1 | 35 |
| 8. Cortez et al. (1989) **16,17** | Laboratory | Anecic  Sp6 | Calcareous alluvial grassland | Grassland | 14 | PF3 | 28 | Wheat straw | 2 – with and without wheat straw | 31 |
| 9. Fisk et al. (2004) **18,19** | Mesocosm in Field | Anecic  Sp3  Mixture SP7 + Sp3 + Sp5 | Acidic Dystrochrepts | Forest | [-2.4;21.8] |  | 0.15 | Forest litter | 2 plots corresponding to 2 EW communities | 275 |
| 10. Lubbers et al. (2015) **20->23** | Laboratory | Endogeic  SP8 + SP7 | Loess soil | Crop | [14 ;18] | θg=0.275 | 0.28-0.51 | Maize residues | 4 (OM surface,or incorporated* 2 types of EW) | 378 |
| 11. Marhan et al. (2007) **24** | Laboratory | Endogeic  SP5 | Stagnic Luvisol | Crop | 20 |  | 1.07 | Wheat straw | 1 | 150 |
| 12. Potthof et al. (2001) **25,26** | Laboratory | Endogeic  SP9 | Orthic Rendzina | Crop | 10 | 50-60% WHC | 4 | Wheat straw | 2- with and without wheat straw | 16 |
| 13. Rizhiya et al. (2007) **27->30** | Laboratory | Anecic  SP2  Endogeic  SP7 | Loamy Typic fluvaquent | Grassland | 16 | θg=0.25 | 0.8/1.95 | Grass | 4 (2 types of EW * 2 bulk densities) | 90 |
| 14. Scheu (1997) **31->39** | Laboratory | Endogeic  SP9 | From Basalt to Limestone soil forest | Forest | 10 |  | 5.45 | No OM  Beech leaf litter  Leaves of stinging nettles | 9 (3 soils*3 OM) | 353 |
| 15. Simek et al. (2010) **40** | Mesocosm in Field | Endogeic  SP8 | Loam-clay Cambisol | Crop | 11 | [0.2-0.25]gw/d DM | 3.93 | 0 | 1 | 147 |
| 16. Snyder et al. (2009) **41** | Laboratory | Epigeic  Sp10 | Haplubrepts | Forest | 18 |  | 5.2 | Leaf litter of oak and hemlock | 1 | 28 |
| 17. Wolters et al. (1993) **42** | Laboratory | Endogeic  SP8 | Rendzinas (Orthic rendzina, FAO) | Forest | 10 | 50% water holding capacity | 9 | Beech leaf litter | 1 | 133 |

(*) Species name (and older name in brackets when different, according Csuzdi, 2012** ) Sp1: Pontoscolex corethrurus (Lumbricus corethrurus); Sp2: Aporrectodea.longa (Allolobophora longa); Sp3: Lumbricus terrestris Sp4: Amynthas tuberculatus (Pheretima tuberculata); Sp 5: Octolasion tyrtaeum (Enterion tyrtaeum) Sp6: Aporrectodea giardi (Allolobophora giardi); Sp7: Lumbricus Rubellus ; SP8: Aporrectodea caliginosa; SP9: Octolasion lacteum (Octolasium lacteum); Sp10: Amynthas Corticis (Perichaeta Corticis)

(**) Csuzdi, Cs. Earthworm species, a searchable database. *Opusc. Zool. Bp*, 43, 97–99 (2012).

Figure S1: Residual frequency histogram obtained from the data base of the 546 pairs of CO_2_ measurements. The obtained Skewness normality index of 0.325 is close to 0 and indicates that normality is satisfied.


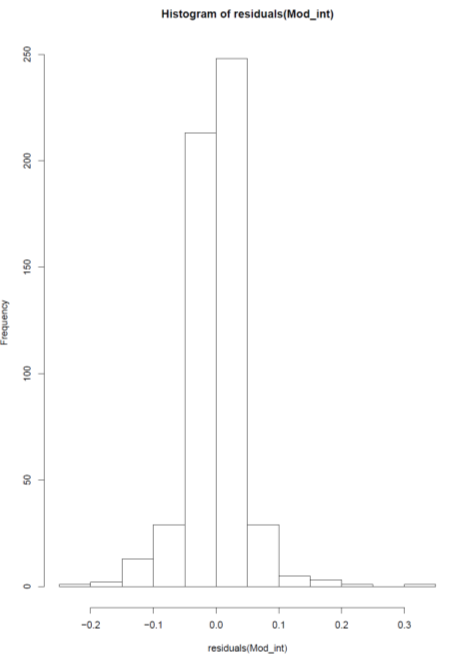

Supplement: Supplementary file 1 — Supplementary Information. [file 41598_2022_17855_MOESM1_ESM.docx]
